# Angiotensin II Activates the Calcineurin/NFAT Signaling Pathway and Induces Cyclooxygenase-2 Expression in Rat Endometrial Stromal Cells

**DOI:** 10.1371/journal.pone.0037750

**Published:** 2012-05-25

**Authors:** Florencia Abraham, Flavia Sacerdoti, Romina De León, Teresa Gentile, Andrea Canellada

**Affiliations:** Instituto de Estudios de la Inmunidad Humoral “Profesor Ricardo A. Margni” (CONICET-UBA), Cátedra de Inmunología, Facultad de Farmacia y Bioquímica, Universidad de Buenos Aires, Buenos Aires, Argentina; National Cancer Institute, United States of America

## Abstract

Cyclooxygenase (COX)-2, the inducible isoform of cyclooxygenase, plays a role in the process of uterine decidualization and blastocyst attachment. On the other hand, overexpression of COX-2 is involved in the proliferation of the endometrial tissue during endometriosis. Deregulation of the renin-angiotensin-system plays a role in the pathophysiology of endometriosis and pre-eclampsia. Angiotensin II increases intracellular Ca^2+^ concentration by targeting phospholypase C-gamma in endometrial stromal cells (ESC). A key element of the cellular response to Ca^2+^ signals is the activity of the Ca^2+^- and calmodulin-dependent phosphatase calcineurin. Our first aim was to study whether angiotensin II stimulated *Cox-2* gene expression in rat ESC and to analyze whether calcineurin activity was involved. In cells isolated from non-pregnant uteri, COX-2 expression -both mRNA and protein- was induced by co-stimulation with phorbol ester and calcium ionophore (PIo), as well as by angiotensin II. Pretreatment with the calcineurin inhibitor cyclosporin A inhibited this induction. We further analyzed the role of the calcineurin/NFAT signaling pathway in the induction of *Cox-2* gene expression in non-pregnant rat ESC. Cyclosporin A abolished NFATc1 dephosphorylation and translocation to the nucleus. Cyclosporin A also inhibited the transcriptional activity driven by the *Cox-2* promoter. Exogenous expression of the peptide VIVIT -specific inhibitor of calcineurin/NFAT binding- blocked the activation of *Cox-2* promoter and the up-regulation of COX-2 protein in these cells. Finally we analyzed *Cox-2* gene expression in ESC of early-pregnant rats. COX-2 expression -both mRNA and protein- was induced by stimulation with PIo as well as by angiotensin II. This induction appears to be calcineurin independent, since it was not abrogated by cyclosporin A. In conclusion, angiotensin II induced *Cox-2* gene expression by activating the calcineurin/NFAT signaling pathway in endometrial stromal cells of non-pregnant but not of early-pregnant rats. These results might be related to differential roles that COX-2 plays in the endometrium.

## Introduction

It has been demonstrated that prostaglandins are involved in the process of uterine decidualization and blastocyst attachment to the uterus. PGE_2_ and PGI_2_ are thought to be implicated in the increase of vascular permeability during implantation and are known to be essential factors for the decidualization process [1], [2]. COX-2 is the inducible isoform of cyclooxygenase, the rate-limiting enzyme that converts arachidonic acid into prostaglandins. The COX-1 isoform is constitutively expressed in most tissues [3], whereas the expression of COX-2 can be induced by several inflammatory stimuli, including cytokines and growth factors. The aberrant expression of COX-2 in the uterine tissue surrounding the blastocyst contributes to the implantation failure in LIF (−/−) mice [5]. COX-2 deficient females are infertile, having abnormalities in ovulation, fertilization, implantation or decidualization [6]. Moreover, overexpression of COX-2 is involved in the proliferation of the endometrial tissue during endometriosis [7]. In spite of this evidence about the relevance of the presence of COX 2 in endometrial tissue, the molecular pathways involved in the regulation of this expression remains unclear.

The expression of COX-2 has been linked to activation of the renin-angiotensin-system (RAS) in cells of the kidney [8]. The RAS is an activation cascade that plays a key role in the regulation of blood pressure and the hydro-electrolytic balance. Renin enzymatically cleaves angiotensin, to produce angiotensin I which in turn is cleaved by angiotensin-converting enzyme (ACE) to render the biologically active effector molecule angiotensin II (Ang II). Ang II acts by binding to types angiotensin (AT)1, AT2, and non-classical- non-AT1/AT2 receptors. During pregnancy, plasma renin concentration and activity as well as Ang II levels are increased [9]. It has been reported that members of the RAS and their receptors play a role in placentation by stimulation of extravillous trophoblast (EVT) invasion [3]. Moreover there is growing evidence indicating that deregulation of both tissue and circulating RAS may be involved in the pathophysiology of pre-eclampsia [9], [10]. In addition, ACE gene polymorphisms were associated with endometriosis development [11].

It has been reported that Ang II increases intracellular Ca^2+^ concentration [Ca^2+^_i_] by interaction with AT1 receptor in trophoblast and in endometrial stromal cells (ESC) [12], [13]. Calcium signaling plays an important role during implantation. The integrin trafficking induced by the ligation of Erb receptors in uterine epithelial and embryonic trophoblast cells is dependent on calcium signaling [14]. Integrin ligation by extracellular matrix fibronectin promotes trophoblast adhesion through the elevation of [Ca^2+^_i_], by targeting phospholypase C-gamma (PLCγ) during mouse blastocyst implantation [15].

A key element of the cellular response to Ca^2+^ signals is the activity of the Ca^2+^- and calmodulin-dependent phosphatase calcineurin (CN) [16]–[18]. The main mechanism of action of this phosphatase characterized so far is the regulation of nuclear factor of activated T cells (NFAT) family of transcription factors. The CN- mediated dephosphorylation promotes translocation of NFAT proteins into the nucleus, where they bind specific elements within target gene promoters, in many cases through association with other transcription factors (reviewed in [19], [20]). The pharmacological action of immunosuppressive drugs such as cyclosporin A (CsA) and FK506 is based on their inhibition of CN in immune effector cells [21].

It has been reported that stimuli inducing a rise in the intracellular calcium concentration are involved in CN/NFAT-mediated induction of COX-2 expression in several cell types [22]–[26] [22]–[26]. In addition, angiotensin II, acting at the AT1 receptor in trophoblast cells, inhibits EVT invasion, via the calcium-activated CN/NFAT signaling pathway [12].

Ang II was related to both, inhibition [27], [28] and induction [29], [30] of COX-2 expression in cells of the kidney. However, whether the RAS has a role in the regulation of gene transcription and COX-2 expression in ESC is not known.

In the present study we have investigated the regulation of *Cox-2* gene expression in endometrial stromal cells. We show that Ang II activates the CN/NFAT signaling pathway in primary cultures of rat ESC, inducing the expression of COX-2 mRNA and protein.

## Results

### COX-2 mRNA and protein expression is transcriptionally induced by CN- dependent calcium signaling in primary ESC isolated from non-pregnant rats

**Figure 1:**
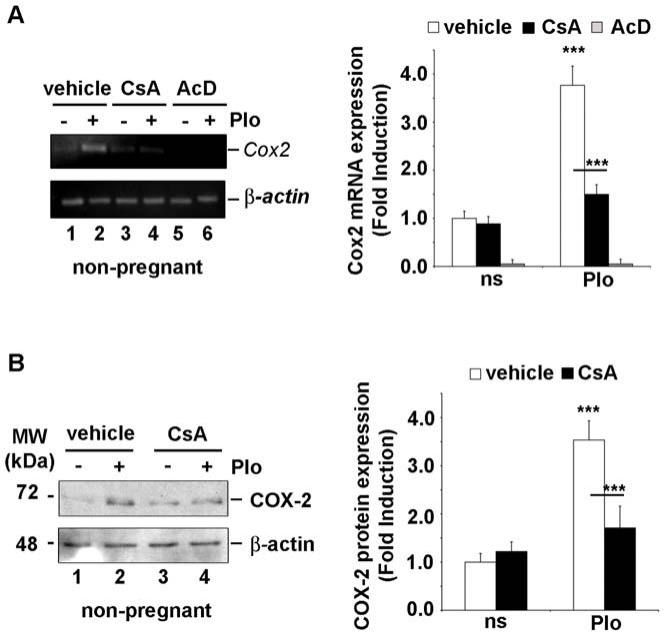
Calcium-induced expression of COX-2 was dependent on calcineurin activity in primary ESC of non-pregnant rats. Primary ESC were isolated from uteri of non-pregnant rats and cultured during 96 h. (A) *Cox-2* mRNA was amplified from total RNA by semi-quantitative RT-PCR. The transcript of *β-actin* was used as internal control. Primary cells were pretreated for 1 h with vehicle (lanes 1–2), 200 ng/ml of CsA (lanes 3–4), or 10 µg/ml of actinomycin D (AcD, lanes 5–6), and then exposed to PIo (a combination of 20 ng/ml of PMA plus 1 µM calcium ionophore Io, for 4 h (lanes 2, 4, 6), or left untreated (lanes 1, 3, 5). (B) Immunoblots of whole extracts obtained from ESC showing endogenous protein expression of COX-2 and β-actin as a loading control. Cells were pretreated as before for 1 h with vehicle (lanes 1–2) or CsA (lanes 3–4) and then exposed to PIo, for 8 h (lanes 2–4). (A and B). Right panels bar plots show the densitometric data analysis of the results shown in the left panels A and B. The COX-2/β-actin ratio was calculated and plotted against the values obtained with the control, non-stimulated rat ESC, which were assigned a value of 1 (ns). The values plotted are the means ± SD of the fold induction values obtained from three independent experiments. Open bars, cells pre-treated with vehicle; closed black bars, cells pre-treated with CsA; closed gray bars, cells pre-treated with AcD. *** P<0.001; ** P<0.01 (ANOVA).

To analyze the regulation of COX-2 expression we isolated ESC from uteri of non-pregnant rats. The isolated cells were cultured during 96 h (until reaching confluence). Contamination of the cultures with epithelial cells was analyzed by assessing expression of vimentin and cytokeratin, by immunocytochemistry. It was found that isolated cells expressed the mesothelial marker vimentin. No cytokeratin-positive cells were detected in any culture (data not shown). The expression of COX-2 was then investigated in primary cultures of the isolated ESC. Cells were cultured in the presence of PMA (20 ng/ml) plus the Ca^2+^ ionophore A23187 (Io, 1 µM) (PIo), a conventional pharmacological means of elevating [Ca^2+^i]. Semi-quantitative RT-PCR revealed a pronounced increase in the expression of *Cox-2* mRNA in primary ESC after treatment with PIo for 4 h; *Cox-2* mRNA was undetected in non-stimulated control cells and no variation was observed in the expression of the housekeeping gene *β-actin* in response to the stimuli (Fig. 1 A). Moreover, it was found that the accumulation of *Cox-2* mRNA was completely inhibited by pre-treatment of cells with the transcriptional inhibitor actinomycin D (AcD, 10 µg/ml, Fig. 1 A, lanes 5 and 6). Pre-exposure of primary ESC cultures to the CN inhibitor CsA (200 ng/ml) potently inhibited the accumulation of *Cox-2* mRNA induced by the PIo calcium stimulus (Fig. 1 A, lanes 3 and 4). The PIo-induced up-regulation of *Cox-2* mRNA expression was paralleled by an increase in the production of COX-2 protein. Immunoblotting assays of whole cell extracts of ESC employing a specific antibody revealed that COX-2 protein was significantly increased in whole extracts from cells treated for 8 h with PIo, compared to levels found in extracts from unstimulated cells (Fig. 1 B, lanes 1 and 2) and was detectable after stimulation for at least 18 h (data not shown). As shown for *Cox-2* mRNA, CsA pre-treatment reduced PIo-induced COX-2 protein synthesis (Fig. 1 B, lanes 3 and 4). Results suggest that PIo stimulation includes a dominant calcium-dependent signaling component which acts via CN.

### Ang II induced CN-dependent-COX-2 mRNA and protein expression in primary ESC isolated from non- pregnant rats

**Figure 2:**
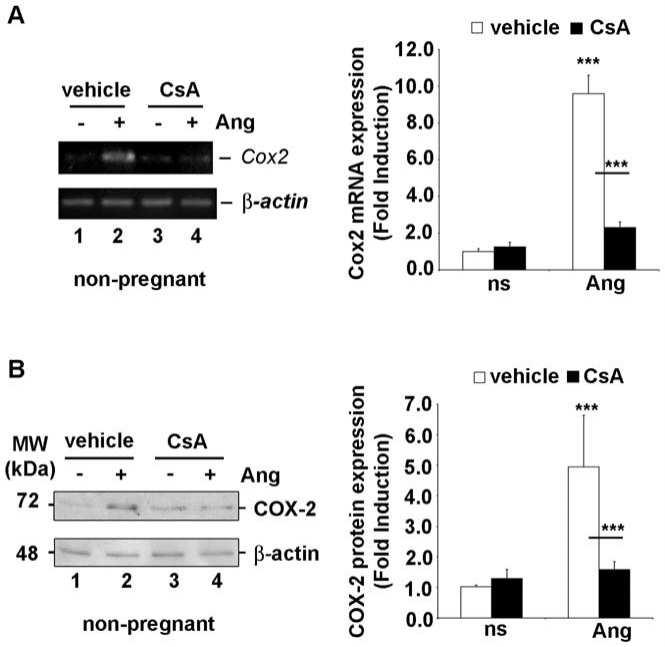
Ang II induced COX-2 expression in primary ESC of non-pregnant rats. Expression was abrogated by CN inhibition. (A) *Cox-2* mRNA was amplified from total RNA purified from primary cultures of ESC, by semi-quantitative RT-PCR. The transcript of the *β-actin* gene was used as internal control. Cells were pretreated for 1 h with vehicle (lanes 1 and 2), or 200 ng/ml of CsA (lanes 3 and 4), and then exposed to 500 nM of Ang II for 4 h (lanes 2 and 4) or left unexposed (lanes 1 and 3). (B) Immunoblots of whole extracts of primary cultured ESC isolated from uteri of non-pregnant rats showing endogenous protein expression of COX-2 and β-actin as a loading control. Primary cultures of the cells were pretreated as before for 1 h with vehicle (lanes 1 and 2), or CsA (lanes 3 and 4), and then exposed to 500 nM Ang II (lanes 2 and 4), or left untreated (lanes 1 and 3) for 8 h. (A and B) Right panel bar plots show the densitometric data analysis of the results shown in the left panels A and B. The COX-2/β-actin ratio was calculated and plotted against the values obtained with the control, non-stimulated rat ESC, which were assigned a value of 1 (ns). The values plotted are the means ± SD of the fold induction values obtained from three independent experiments performed. Open bars, cells pre-treated with vehicle; closed black bars, cells pre-treated with CsA *** P<0.001; ** P<0.01 (ANOVA).

ESC isolated from non-pregnant rat uteri were cultured in the presence of Ang II. Employing semi-quantitative RT-PCR, it was found that Ang II stimulation (500 nM) during 4 h induced *Cox-2* mRNA expression in primary ESC isolated from non-pregnant rats (Fig. 2 A, lanes 1 and 2). Inhibition of CN by pre-treatment with CsA, significantly inhibited the synthesis of *Cox-2* mRNA in ESC (Fig. 2 A, lanes 3 and 4).

The expression of COX-2 protein was then examined in whole extracts of ESC by western blot. Likewise PIo, Ang II stimulation during 8 h upregulated COX-2 protein levels in ESC isolated from non-pregnant rats (Fig. 2 B, lanes 1 and 2). Pre-treatment of ESC with CsA, diminished significantly the COX-2 protein expression (Fig. 2 B, lanes 3 and 4).

### NFAT expression and Ca^2+^ activation of the CN/NFAT pathway in ESC

The pharmacological stimulation of ESC isolated from non-pregnant rats with agents that induce a rise in the cytoplasmic calcium concentration, induced an increase of mRNA and protein expression of COX-2 and this effect was significantly reduced when cells were pretreated with CsA. In order to determine whether the NFAT transcription factor is involved in the PIo and/or Ang II stimulation of COX-2 expression, the activation of the CN/NFAT pathway in the cells was analyzed.

**Figure 3:**
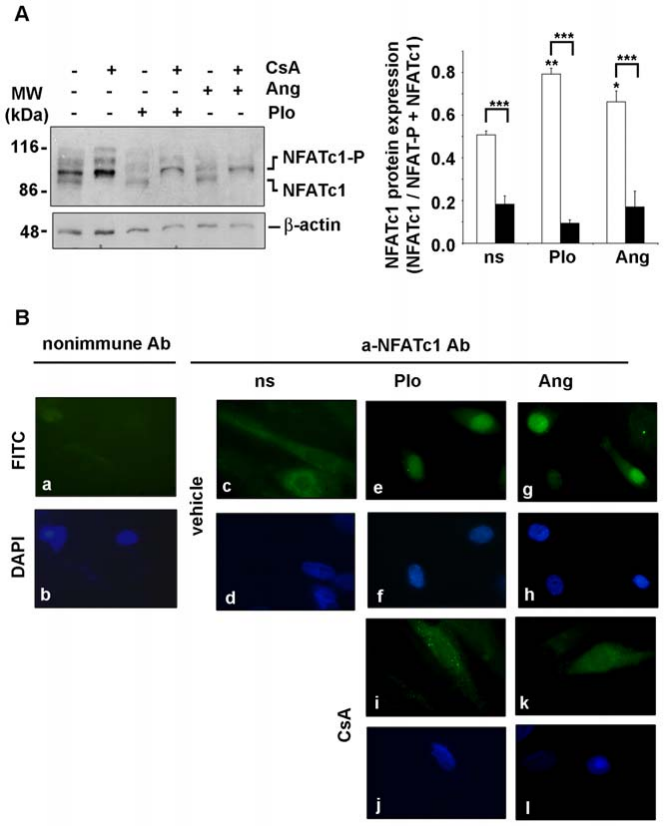
NFAT dephosphorylation and nuclear localization was abrogated by CN inhibition in primary rat ESC. (A) Immunoblots of whole extracts of primary cultured ESC isolated from uteri of non-pregnant rats showing endogenous expression of NFATc1 and β-actin as loading control. Primary cultures of cells isolated from uteri of non-pregnant rats were pretreated for 1 h with vehicle (lanes 1,3, 5) or 200 ng/ml of CsA (2, 4, 6), and then exposed for 2 h to PIo (lanes 3 and 4); 500 nM of Ang II (lanes 5 and 6), or were left unstimulated (ns, lanes 1 and 2). The position of phosphorylated NFAT (P-NFATc1) and dephosphorylated NFATc1 (NFATc1) is indicated. Right panel bar plot shows the densitometric data analysis of the results shown in the left panel. The NFAT/β-actin ratio was calculated and then the NFATc1/(NFATc1+NFATc1-P) ratio was plotted. The values are the means ± SD obtained from three independent experiments performed. Open bars, cells pre-treated with vehicle; closed black bars, cells pre-treated with CsA ** P<0.01; * P<0.05 (ANOVA). (B) Immunofluorescence analysis of endogenous NFAT protein with anti-NFATc1 antibody (c–l) or nonimmune Ab (a and b) as control of unspecific staining. Primary cultures of cells isolated from non-pregnant rats were pretreated for 1 h as before with vehicle (c–h) or CsA (i–l), and then exposed 2 h to PIo (e–f, i–j); 500 nM of Ang II (Ang, g–h, k–l), or were left unstimulated (ns, c–d). (a, c, e, g, i, k): FITC staining of the cells. (b, d, f, h, j, l): nuclei staining with DAPI. Magnification 200×. Shown is a representative experiment out of three performed.

The expression, phosphorylation status and subcellular localization of NFATc1, in PIo and Ang II stimulated ESC was evaluated. Total extracts from non-stimulated and stimulated ESC were analyzed by immunoblot with a specific antibody against NFATc1. As shown in Fig. 3 A, in cells pre-treated with CsA for 1 h, the inhibition of NFAT-protein dephosphorylation was evident in the retardation of the protein bands recognized by the antibody (Fig. 3 A lanes 2, 4 and 6). It is noteworthy that the mobility of NFAT protein was very similar in non-stimulated and stimulated cells (Fig. 3 A lanes 1, 3 and 5). Nevertheless densitometric analysis revealed that stimuli induced dephosphorylation of NFAT, as resulted from compared dephosphorylated/total NFAT ratio between stimulated and non stimulated cells (Fig. 3 A, open bars). These data were confirmed by immunofluorescence. NFATc1 nuclear immunostaining was mainly observed in PIo or Ang II-stimulated cells (Fig. 3 B). Nuclear localization of NFATc1 was evident after 30 min of PIo as well as Ang II exposure. In cells pretreated with CsA, staining of the nucleus diminished and NFATc1 immunoreactivity was mainly in the cytoplasm of the cells.

### The CN/NFAT signaling pathway is required for calcium-dependent activation of Cox-2 gene expression in ESC isolated from non-pregnant rats

Results demonstrated that CN is involved in induction of COX-2 expression in ESC isolated from non-pregnant rats. Furthermore, it was demonstrated that the NFATc1 is dephosphorylated and it is mainly located in the nucleus of PIo and Ang II- stimulated ESC.

**Figure 4:**
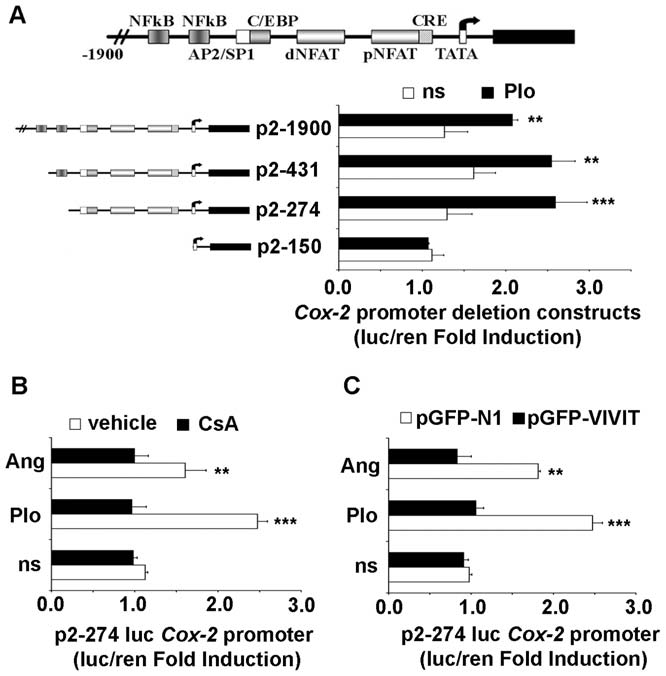
Inhibition of CN and endogenous CN-NFAT binding blocked *Cox-2* gene promoter activation in primary ESC. (A) ESC isolated from uteri of non-pregnant rats were transiently transfected with a series of luciferase reporter plasmids containing regions of the human *Cox-2* promoter starting from −1900 down to −150 upstream of the transcription initiation site. The schematic representation of the proximal 1900 bp region of the human *Cox-2* gene promoter, showing the positions of putative transcription factor response elements [22] is embedded. Cell cultures were co-transfected with *Renilla* plasmids to normalize for transfection efficiency. Transfected cells were treated for 4 h with vehicle (ns, open bars) or PIo (solid bars), and the luciferase activity determined in cell lysates. Transcriptional activity is expressed as the fold increase in luciferase activity above baseline levels from transfected, nonstimulated control cells. (B) ESC transfected with the −274 *Cox-2* luciferase reporter construct were pretreated for 1 h with vehicle (open bars) or 200 ng/ml of CsA (solid bars) and treated for 4 h with PIo or 500 nM of Ang II as indicated. Data are presented as in A. (C) Primary stromal cells isolated from uteri of non-pregnant rats were transfected with 800 ng of expression constructs encoding either pEGFP-VIVIT (solid bars) or pEGFP-N1 as control (open bars). Transfected cells were stimulated as before for 4 h with PIo, Ang II, or left untreated (ns), and the luciferase activity determined in cell lysates. Data are presented as in A. (A–C) Results shown are from a representative experiment of three performed, and values are the means ±SD of triplicate determinations. *** P<0.001; ** P<0.01 (ANOVA).

To evaluate the participation of NFATc1 in the calcium- induced COX-2 gene expression in ESC isolated from non-pregnant rats, we first studied whether calcium signals activate transcription of the human *Cox-2* promoter in these cells. ESC were transfected with luciferase reporter constructs; these constructs were driven by versions of the human *Cox-2* promoter harboring nested deletions in the region spanning between −1900 bp to +2 bp from the TATA box (Fig. 4 A and [23]). Constructs containing the 274 bp upstream of the TATA box (−1900/+2, −431/+2, and −274/+2) supported a PIo -induced luciferase activities that was 2–3 fold greater than that observed in non-stimulated cells (Fig. 4 A). In contrast, the construct containing only the proximal 150 bp promoter region (−150/+2) did not support increased luciferase activity above non-stimulated levels. The latter results are in line with reports indicating that two NFAT binding sites located between base pairs −274 and −150 of the human *Cox-2* promoter are required for induction via the calcium/CN pathway [22], [23], [26]. PIo and Ang II-dependent activation of the p2–274 *Cox-2* luciferase reporter construct was inhibited by pre-treatment of cells with CsA (Fig. 4 B).

We confirmed that NFAT transcription factors participate in the regulation of the *Cox-2* promoter in ESC by inhibiting endogenous NFAT signaling with a GFP fusion protein bearing the VIVIT peptide (GFP-VIVIT). The VIVIT peptide specifically inhibits the CN/NFAT pathway by blocking the binding of calcineurin to NFAT proteins, thereby preventing NFAT de-phosphorylation [31]. Expression of GFP-VIVIT effectively inhibited the PIo as well as Ang -induced activity of the p2–274 *Cox-2* luciferase reporter construct (Fig. 4 C), whereas GFP alone had no effect.

**Figure 5:**
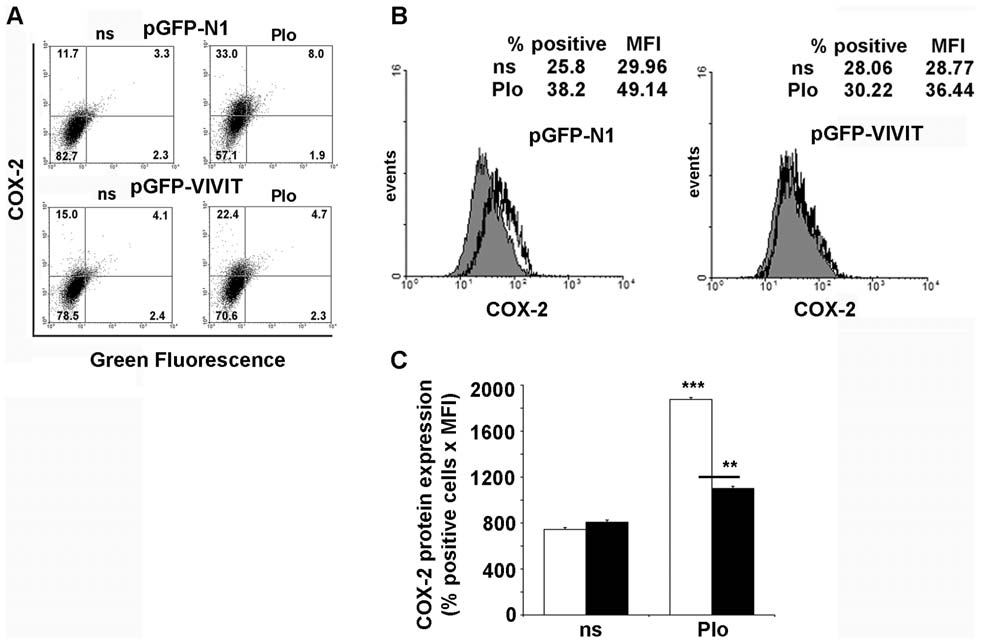
Inhibition of endogenous CN-NFAT binding with the exogenously expressed VIVIT peptide blocked COX-2 protein expression. (A and B) Primary stromal cells isolated from uteri of non-pregnant rats were transfected with 800 ng of expression constructs encoding either pEGFP-VIVIT or pEGFP-N1 as control. Transfected cells were stimulated for 14 h with PIo, or left untreated (ns). Cells were permeabilized and stained for COX-2. Detection of cells co-expressing both COX-2 and GFP protein was analyzed by two color-FACScan. (A) Density plots showing FACScan analysis of transfected cells immunostained for COX-2 protein. The percentage of acquired cells in each quadrant is embedded. (B) Upper panels: Histograms showing COX-2 immunostaining of gated, GFP positive- cells. Left: pGFP-N1- transfected cells; rigth: pGFP-VIVIT- transfected cells. Closed gray histogram: non-stimulated (ns) cells; open black histogram: (PIo)- stimulated cells. The percentage of COX-2-positive cells (% positive) and the mean fluorescence intensity (MFI) from each histogram are enclosed. Lower panel: Bar graphs showing the product of the percentage of COX-2-positive cells and the mean fluorescence intensity, from GFP-positive cells ploted in upper panels. Open bars: cells transfected with pEGFP-N1; dark bars: cells transfected with the pEGFP-VIVIT. (A–B) The results shown are from a representative experiment of three performed, and values are the means ±SD of duplicate determinations. *** P<0.001; ** P<0.01 (ANOVA).

To further determine the role of NFAT in the Ca^2+^- induced COX-2 expression we tested whether COX-2 protein expression in ESC could also be inhibited by the selective peptide inhibitor of NFAT, VIVIT. The plasmids encoding GFP and the fusion protein GFP-VIVIT were introduced in the ESC. The day after transfection, cells were stimulated with PIo. COX-2 production was analyzed by intracellular staining of COX-2 which permitted direct comparison of COX-2 production by transfected (GFP-positive) and nontransfected (GFP-negative) cells at a single cell level in the same sample. Data analysis revealed that in GFP-N1 transfected cells, PIo induced an increase in the number of COX-2 positive, GFP expressing cells (Fig. 5 A, upper right quadrants in upper panels). In contrast, this increase was not observed in GFP-VIVIT transfected cells (Fig. 5 A, upper right quadrants in lower panels). The inhibitory effect of GFP-VIVIT on COX-2 production in this cell population was reflected in the decreased number of COX-2- producing cells (% positive) and in the mean fluorescence intensity (MFI) of these cells. The product of these 2 numbers (% positive×MFI) is a measure of total COX-2 production by the GFP or GFP-VIVIT–expressing cells (Fig. 5 B and [32]) in non-stimulated and PIo-stimulated cells. The percentage of inhibition by VIVIT of the PIo-induced COX-2 expression was 74%, showing the dependence of COX-2 production on NFAT.

### Induction of COX-2 mRNA and protein is not dependent on CN activity in primary ESC isolated from pregnant rats

**Figure 6:**
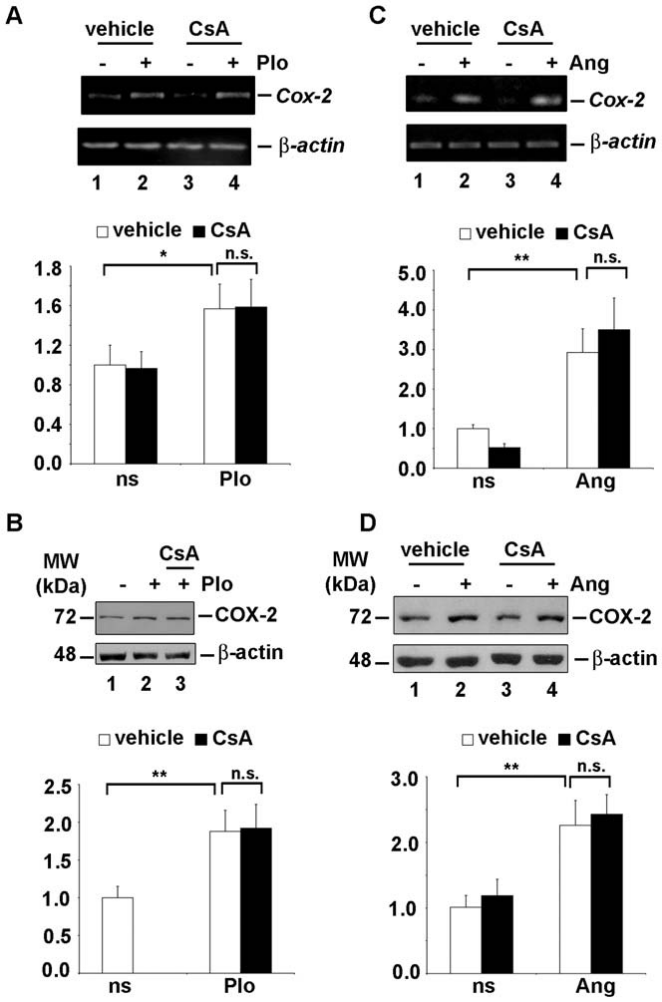
Calcium-induced COX-2 expression was not abrogated by CN inhibition in primary ESC from early pregnant rats. ESC were isolated from uteri of pregnant rats on 4.5 day post coitus (d.p.c). (A and C) The *Cox-2* mRNA was amplified from total RNA purified from primary cultures of the isolated cells by semi-quantitative RT-PCR. The transcript of *β-actin* was used as internal control. Cells were pretreated for 1 h with vehicle (lanes 1 and 2) or 200 ng/ml of CsA (lanes 3 and 4), and then exposed for 4 h to PIo (A), 500 nM of Ang II (C) (lanes 2 and 4) or left unstimulated (lanes 1 and 3). (B and D) Immunoblots showing endogenous protein expression of COX-2 and β-actin as a loading control. (B) Primary cultures of the isolated cells were pretreated for 1 h as before with vehicle (lanes 1 and 2) or CsA (lane 3), and then exposed to PIo for 8 h (lanes 2 and 3) or not (lane1). (D) Primary cultures of the isolated cells were pretreated for 1 h with vehicle (lanes 1 and 2) or CsA (lane 3 and 4), and then exposed to Ang II for 8 h (lanes 2 and 4) or not (lanes1 and 3). (A–D) Lower panels bar graphs show the densitometric data analysis of the results shown in upper panels. The COX-2/β-actin ratio was calculated and plotted against the values obtained with the control, vehicle-treated non-stimulated rat ESC (ns), which were assigned a value of 1. Open bars, cells pre-treated with vehicle; closed black bars, cells pre-treated with CsA. Values plotted are the means ± SD of the fold induction values obtained from three independent experiments. *P<0.05; ** P<0.01 (ANOVA).

The expression of COX-2 was then investigated in primary cultures of ESC isolated from pregnant rats. Similar to a previous report [33], semi-quantitative RT-PCR analysis and immunoblot assays revealed that COX-2 mRNA and protein expression in ESC increases significantly on days 4.5 and 10.5 of early pregnancy compared to the transcript levels found in cells isolated from non-pregnant rats (data not shown). Semi-quantitative RT-PCR analysis showed that *Cox-2* mRNA was further upregulated by PIo (Fig. 6 A) and by Ang II stimulation during 4 h (Fig. 6 C) in ESC isolated at 4.5 days of early pregnancy. Pre-treatment of cells with CsA did not affect the *Cox-2* mRNA accumulation (Fig. 6 A and C, lanes 3 and 4). No variation was observed in the expression of the housekeeping gene *β*-*actin* in response to the stimuli or the inhibitor. Consistent with the results on mRNA transcription, induction of COX-2 protein in primary ESC isolated at 4.5 days of pregnancy, was not affected by CsA-treatment of the cells before PIo and Ang II stimulation during 8 h (Fig. 6 B and D, lanes 3 and 4). These results suggest that, unlike the non-pregnant status, a CN independent component of stimuli is involved in the PIo and Ang II- induced upregulation of COX-2 in ESC isolated from pregnant rats.

## Discussion

The relevance of COX-2 expression in the uterus has been established [6]. It has been reported that COX-2 expression during the pre-implantation period is involved in the decidualization process, by mediating PGE_2_ synthesis [34]. PGE_2_ could also be involved in the regression of decidua basalis, since it is significantly increased during this period of pregnancy in the rat uterus, with COX-2 mediating its induction [33]. It has been reported that the increased COX-2 expression and PGE_2_ synthesis can mediate aromatase expression and estradiol synthesis in eutopic as well as in ectopic ESC of patients with endometriosis. COX-2 in turn is stimulated by estradiol, and PGE2 itself, thus establishing a positive feedback loop that favors continuous formation of E2 and PGE2 in endometriosis (reviewed in [7] and [35]). Induction of COX-2 expression was also observed in the syncytiotrophoblast [36] as well as in endothelium and smooth muscle in systemic vasculature [37] of preeclamptic patients, and COX-2 expression was related to the reduced blood flow in those patients. Furthermore, it has been established the role played by COX-2 during carcinogenesis. Aberrant COX-2 overexpression was consequently found in various human malignomas like breast, prostate, bladder, pancreas, skin, lungs and gastrointestinal tract carcinomas [38], more recently also hematological malignancies [39]. Moreover, studies in vivo and in vitro confirmed the role of COX-2 in the development of ovarian and cervical cancer which were associated with an increase in angiogenesis markers [40], [41]. Therefore, a detailed understanding of the signaling network controlling COX-2 expression is necessary for better characterization of the differentiation process in endometrium and for its proper application in reproductive biology. Recently, St. Louis and co-workers [42] have studied the expression, activity and regulation of COX-1 and COX-2 by sex steroids at specific stages in the pregnant, pseudo-pregnant and non-pregnant rat uterus. However, the signal transduction pathways involved in such regulation were not elucidated. The present study demonstrates the calcium activated CN/NFAT dependent induction of the *Cox-2* gene expression in ESC.

We observed that the *Cox-2* gene was transcriptionally induced by phorbol ester plus a calcium ionophore (PIo) in primary cultures of ESC. PIo is a stimulus widely used to rise intracellular Ca^2+^ concentration [Ca^2+^ i] and thereby triggering intracellular signaling pathways such as CN/NFAT. In cultures of ESC isolated from non-pregnant animals, the COX-2 induction was abolished by pretreatment with the CN inhibitor CsA, similar to that observed in other cell types [22], [23], [26]. As in other cell types, stimuli that only augment [Ca^2+^ i] such as calcium ionophore, are not sufficient to full induce COX2 expression (data not shown and [22], [23], [26]). This is consistent with the known tendency of NFATs to cooperate with other transcription factors, including AP1, GATA 4, MEF/2 and C/EBP and thereby integrate diverse signaling pathways [19], [20]. Many of the factors that form transcription complex with NFATs are regulated by PKC and RAS/MAPK pathways that are triggered by the use of phorbol esters such as PMA [43].

It has been reported that Angiotensin II, acting at AT1 receptors, induced COX-2 expression in thick ascending limb cells of Henle's loop and in glomerular mesangial cells of the kidney [8], [30]. Ang II is known to activate calcium signaling in different cell types, including trophoblast and ESC [12], [13], [44], [45]. In trophoblast cells Ang II, acting at the AT1 receptor, inhibits EVT invasion, via the calcium-activated CN/NFAT signaling pathway [12]. In rat-uterine stromal cells Ang II induced in vitro the PGE_2_-dependent activation of the decidual reaction [46]. In line with these results, we found induction of the *Cox-2* gene in ESC stimulated with Ang II. Furthermore, in ESC isolated from non-pregnant rat uterus this induction was dependent on CN activity.

Regardless of the animal species, the *Cox-2* promoter contain a classical TATA box, an E-box, and binding sites for transcription factors such as nuclear factor kB, nuclear factor-IL6/CCAAT enhancer binding protein and cyclic AMP-response element (CRE) -binding proteins. These sequences have been shown to act as positive regulatory elements for the *Cox-2* gene transcription in various cell types [47]–[50]. PMA as well as Ang II can trigger NF-κB, C/EBP, and AP1/CREB pathways, thus promoting *Cox-2* gene transcription. [51]–[53]. Moreover calcium- activated CN is known to bind members of the NFAT transcription factors, inducing their dephosphorylation, nuclear translocation, and the transcriptional activation of the *Cox-2* gene in several cell types [22], [23], [26], [54]. Ang II Receptor (AT)1-dependent activation of the CN/NFAT signaling pathways have been described in placenta and trophoblast cells [12], [55], however, whether Ang II activates this signaling pathway in uterine stromal cells have remained unknown so far. A previous study has revealed CN A and NFATc1 mRNA and protein expression in the uterus of pregnant mice at terminus [56]. These authors found, by quantitative RT-PCR analysis of whole uterine RNA, that message of CN A1 as well as NFATc1, c2 and c4 were expressed in the mouse uterus, and these levels were shown to increase during the course of pregnancy. In addition, we found that NFATc1 protein is expressed in primary cultures of ESC from non-pregnant (Fig. 5) and pregnant rats (data not shown). Moreover, the pretreatment of cells with CsA for 1 h inhibited NFAT-protein dephosphorylation, and its nuclear localization in PIo or Ang II stimulated cells.

Promoter deletion experiments performed in primary cultures of ESC isolated from uteri of non-pregnant rats, located the CN-dependent induction of *Cox-2* in the region between base pairs −274 and −150. This region contains two NFAT binding motifs and it is involved in the regulation of *Cox-2* expression in several cell types treated with stimuli that increased intracellular Ca^2+^ concentrations [22], [23], [26], [54]. This region also contains an AP1- like site adjacent to the proximal NFAT binding motif [22]. It has been found previously that PKC activators, such as PMA, induced COX-2 expression in ESC [57]. Those PKC activators contribute to the activation of several transcription factors including NF-κB, AP-1, and octamer binding factors [58]. In this work we confirmed the CN/NFATc1- dependent activation of *Cox-2* gene expression in ESC by transient over-expression of the peptide VIVIT, a specific inhibitor of the NFAT binding to CN, in primary ESC isolated from non-pregnant rats, thus indicating that CN/NFAT interaction is required for the full induction of *Cox-2* gene transcription in these cells. However whether NFAT requires cooperation with other transcription factor such as AP-1 to full induction of COX-2 transcription in ESC deserves further investigation.

It has been reported that IL-8 and IL-11 are induced by the CN/NFAT signaling pathway in prokineticin 1 receptor (PROKR1)- and prostanoid F receptor-expressing Ishikawa (human endometrial adenocarcinoma) cells [59]–[62]. In those papers the authors have described PROK1- and Prostaglandin F2 alpha-induced cytokine expression also in first trimester decidua, but not CN/NFAT expression and activation nor the participation of this pathway in the transcriptional regulation of the cytokines in this tissue. Thus, the present study is the first description of the CN/NFAT-dependent regulation of gene expression in primary normal ESC.

In agreement with previous studies [33], [42], we found a significant increase in COX-2 mRNA and protein expression in primary cultures of unstimulated ESC isolated from early pregnant rats compared to levels found in non-pregnant rats (data not shown). In pregnant rat ESC, PIo and Ang triggered further COX-2 induction; however, this induction was not inhibited by CsA, strongly suggesting that CN activity is not involved. Although it was not demonstrated in this work, the NF-κB activation in the endometrium during early pregnancy described so far [63], [64] may account for the differential regulation of COX-2 expression in ESC between non-pregnant and pregnant uteri. In addition it has been reported that low levels of progesterone in early pregnancy are associated with the activation of the NF-κB signaling pathway and the induction of gene transcription of inflammation-related molecules including COX-2 [65].

In conclusion, the main goal of this study was to describe for the first time the transcriptional regulation of the *Cox-2* gene by Ang II and the calcium-activated CN/NFAT signaling pathway in primary cultures of normal endometrial stromal cells. The COX-2 mediated synthesis of prostaglandins is a major step in the increased angiogenesis observed during normal pregnancies and gynecological diseases. It was described that CN/NFAT- activated COX-2 expression was involved in the VEGF-induced angiogenesis in the cornea [23]. Thus, it will be of great interest to identify the roles of the calcium/CN/NFAT pathway in the different physiological and pathological processes in the female reproductive tract. Targeted disruption of NFAT members should be made in order to evaluate its role during uterine differentiation *in vivo*.

## Materials and Methods

### Animals and protocols

Adult female and male Sprague Dawley rats (150–200 g weight) were maintained at the Institute animals facilities in a 14 h light–10 h darkness photoperiod, and controlled room temperature (21±4°C). All procedures were conducted under consent of the Committee on the Ethics of animal experiments of “Instituto de Estudios de la Inmunidad Humoral Prof. R. Margni” (Permission number: 3/2011), in accordance with guidelines of EU Directive 2010/63/EU, and AADEAL (Asociación Argentina de Especialistas en Animales de Laboratorio) recommendations for experiments involving animals. Animals were provided with a pelleted diet and water *ad libitum*. Females were mated at proestrus with male rats. The presence of the vaginal plug was considered as day 0.5 of pregnancy. Animals were killed by CO_2_ inhalation in the morning of days 0 (non-pregnant) and 4.5 of pregnancy, uteri were excised aseptically and subjected to enzyme digestion to isolate uterine cells. Five rats were used for each time of pregnancy.

### Reagents

Phorbol 12-myristate 13-acetate (PMA), the Ca^2+^ ionophore A23187 (Io), Angiotensin II (Ang II), and actinomycin D (ActD), were all purchased from Sigma Chemical (St. Louis, MO). Cyclosporin A (CsA) was purchased from Molecular Bio Products (San Diego, CA).

### Isolation and culture of endometrial stromal cells (ESC)

Uteri from 5 rats per group were removed and pooled. ESC-enriched uterine cells were separated from epithelial cells by sequential enzyme digestion as described [66]. ESC-enriched uterine cells suspensions were resuspended in Dulbecco's Modified Eagle Medium (D-MEM)/Ham F-12 nutrient mixed 1∶1 (Gibco-BRL/Invitrogen) containing 10% fetal bovine serum (FBS; Gibco-BRL/Invitrogen) supplemented with 20 mM Hepes, 100 µg/ml streptomycin, 100 U/ml penicillin, and 2 mM L-glutamine (all from Gibco-BRL/Invitrogen), and plated into 100 mm-diameter culture dishes during 1 h to allow macrophages and granulocytes to adhere to the dish. Unattached cells were recovered and re-plated at 5×10^5^/1.5 ml per well in 6-well plates, and cultured at 37°C and 5% CO_2_ in an humidified incubator to allow ESC to adhere to plates. After 24 h, media was replaced and cells were cultured in complete medium with 48 h-interval changes of culture medium to remove non-adherent cells present in the supernatant, until ESC reached confluence (1 week). The quality of the stromal cell preparation was assessed by immunocytochemical detection of cytokeratin and vimentin, as described below.

### Immunofluorescence

ESC were plated on glass coverslips and cultured in a 24 well culture plate, as described above. To assess quality of the stromal cell culture, cells were washed twice with cold PBS and fixed for 15 min with 4% paraformaldehyde (w/v) in PBS. Cells were washed with PBS and permeabilized for 10 min with PBS containing 0.25% (v/v) Triton X-100. After three washes with PBS, fixed cells were blocked with 10% bovine serum albumin in PBS (PBS/BSA) for 20 min. Cells were incubated for 1 h at room temperature with either a mouse monoclonal anti-vimentin antibody (Sigma), or a monoclonal anti-cytokeratin antibody (Sigma). After three washes with PBS, cells were incubated for 30 min at room temperature with the secondary antibody (FITC-labeled goat anti-mouse IgG, Molecular Probes, Eugene, OR). Cells were mounted in a DAPI-containing mounting media (Molecular Probes) and analyzed by fluorescence microscopy (Axiophot, Carl Zeiss, Jena, Germany).

To determine nuclear localization of NFATc1, ESC cultured on coverslips were deprived of FCS during 16 h. Cells were exposed to vehicle or inhibitor (CsA, 200 ng/ml) for 1 h and then treated with the pharmacological stimuli: PIo, a combination of 20 ng/ml phorbol myristate acetate (PMA) and 1 µM calcium ionophore (Io); Ang II (500 nM) for 2 h. Cells were then washed, fixed, permeabilized and blocked as described above and then incubated for 1 h at room temperature with a mouse monoclonal anti-NFATc1 antibody (clone 7A6, Santa Cruz Biotechnology, Santa Cruz, CA). After washing, cells were incubated with the secondary antibody (FITC-labeled goat anti-mouse IgG, Santa Cruz Biotechnology), mounted and analyzed by fluorescence microscopy, as described above.

### Cell lysis and Immunoblot analysis

Cells were grown in 6-well plates and cultured *in vitro* without FCS supplementation during 16 h before experiments. Cell were exposed to vehicle or inhibitor (CsA, 200 ng/ml) for 1 h and then treated with the pharmacological stimuli. After 8 h of incubation, whole-cell extracts were obtained as previously described [67]. The protein content in the extracts was determined by the Bradford's method [68]. Total extracts were then boiled in Laemmli's buffer and 25 µg of the protein mixture were then resolved by 10% SDS-PAGE (8% polyacrylamide for NFATc1), under reducing conditions. Proteins were transferred to nitrocellulose membranes that were then blocked overnight at 4°C in Tris-buffered saline plus 0.1% Tween-20 (TBST) containing 5% (w/v) skimmed milk. Membranes were probed with the following antibodies: a goat anti-rat COX-2 polyclonal antibody, a goat anti- rat β-actin, polyclonal antibody and a mouse anti-NFAT c1 monoclonal antibody (clone 7A6, all from Santa Cruz Biotechnology). Membranes were then incubated with a peroxidase-labeled secondary antibody and bound antibodies were detected by the ECL western blotting analysis kit (Pierce, Thermo Fisher Scientific, Rockford, IL).

### RNA Isolation, Reverse Transcription, and PCR Analysis

Cells were grown in 6-well plates and cultured *in vitro* without FCS supplementation of cultured medium during 16 h before experiments. Cultured rat ESC were exposed to inhibitors (CsA, 200 ng/ml; actinomycin D, 10 µg/ml), and the pharmacological stimuli. Total RNA was isolated from cells with TriZol isolation reagent (Invitrogen-Life Science, Grand Island, NY). We performed electrophoresis of RNA samples in 2% agarose gels to check for genomic DNA contamination and also for RNA degradation. Transcripts encoding rat *Cox-2* were analyzed by semiquantitative RT-PCR. One µg of total RNA was reverse-transcribed to cDNA. The cDNA obtained was then used for PCR amplification with specific primers for rat *Cox-2* or *β-actin*: *Cox-2* forward primer, 5′-ACTTGCTCACTTTGTTGAGTCATTC-3′; reverse primer, 5′-TTTGATTAGTACTGTAGGGTTAATG-3′; *β-actin* forward primer, 5′-GTCGACAACGGCTCCGGCA-3′; reverse primer, 5′-GTCAGGTCCCGGCCAGCCA-3′. PCR reactions were carried out as previously described [26]. Negative controls in which cDNA sample was absent from PCR reaction mixture were made. Amplified cDNAs were separated by agarose gel electrophoresis, and bands were visualized by ethidium bromide staining. Data shown correspond to the number of cycles where the amount of amplified product is proportional to the abundance of starting material.

### Plasmid Constructs and Transient Transfection Assays

The GFP-VIVIT construct encodes an N-terminal fusion of the high affinity calcineurin-binding peptide (VIVIT) to GFP protein [31]. The pEGFP-N1 expression vector was purchased from Clontech Laboratories, Inc. (Mountain View, CA). The *Cox-2* (p2–1900) luc plasmid containing the human *Cox-2* promoter and the derived deletion constructs *Cox-2* (p2–431) luc; *Cox-2* (p2–274) luc; and *Cox-2* (p2–150) luc, [22] were kindly provided by Dr. Manuel Fresno (Centro de Biología Molecular Severo Ochoa, Madrid, Spain). Null *Renilla* was purchased from Promega (Madison, WI).

ESC were plated on 35-mm dishes at 90% confluence the day before transfection. Cells were transfected with the FuGene HD reagent (Roche Applied Science, Buenos Aires, Argentina) in complete DMEM-Ham F12 medium. Two µg of plasmid DNA were used per well. Individual transfections were made up to 2 µg with empty vector. Transfected cells were incubated for 24 h at 37°C and 5% CO_2_. At the end of the transfection period, culture medium was removed and replaced by fresh DMEM:Ham F12 plus 10% fetal bovine serum and cells were further incubated for 24 h. Cells were incubated during 16 h without FBS supplementation of culture medium and then exposed to the pharmacological stimuli. For the luciferase reporter experiments, after 5 h stimulation, cells were lysed according to the instructions of the Dual Glo Luciferase assay kit (Promega), and luciferase activity was measured in a luminometer (Victor Multilabel Plate Reader, Perkin Elmer, Waltham, MA). All samples were tested in triplicate, and the results were normalized to a *Renilla* luciferase internal control. For COX-2 protein expression assays the stimulated cells were harvested after 14 h and analyzed by flow cytometry, as described below.

### Flow cytometry

Expression of COX-2 protein was assessed in GFP-VIVIT as well as in pEGFP-N1 transfected cells by flow cytometry. Briefly, transfected cells were exposed to PIo for 14 h or left unstimulated (ns). Cells were harvested by treatment with Trypsin/EDTA (Gibco-BRL/Invitrogen), washed twice with cold PBS and fixed for 10 min with 0.01% paraformaldehyde (w/v) in PBS. Cells were washed three times with PBS and permeabilized for 15 min with PBS containing 0.5% (v/v) saponin. After three washes with PBS-0.1% saponin (PBS-S), fixed cells were blocked with 10% FBS in PBS-S containing 1% sodium azide for 20 min. Cells were washed twice with PBS-S and then cells were incubated for 1 h at room temperature with a goat polyclonal anti-COX-2 antibody (Santa Cruz Biotechnology). After three washes with PBS-S, cells were incubated for 30 min at room temperature with the secondary antibody (Alexa 633-labeled rabbit anti-goat IgG, Molecular Probes). Cells were washed twice in PBS-S, resuspended in ice cold PBS-S/BSA/sodium azide, and acquired by a Cell cytometer (Partec-Pas III, Görlitz, Germany). Data were analyzed using the WinMDI 2.9 free software (http://facs.scripps.edu/software.html)

### Data analysis

The COX-2/β-actin ratios were calculated from the densitometric data analysis of the RT-PCR and immunoblotting assays. The NFATc1/β-actin ratios were calculated from the densitometric data analysis of the immunoblotting assays. The values plotted are the means ± SD of the fold induction values obtained from three independent experiments. Data of luciferase reporter assays and flow cytometry are the means ± SD of triplicate determinations from one representative experiment of three performed.

Differences between groups were tested for significance using one-way analysis of variance (ANOVA), and the Student-Newman-Keuls multiple comparison test or the Bonferroni test (Fig. 3A) as post-test. (***) corresponds to a significance of P<0.001, (**) corresponds to P<0.01, (*) to P<0.05 and (n.s), not significant, corresponds to P>0.05. (Motulsky, HJ Prism 4 Statistics Guide. Graph-Pad Software Inc., San Diego CA 2003).

## References

[1] Kennedy TG (1980). Estrogen and uterine sensitization for the decidual cell reaction: role of prostaglandins.. Biol Reprod.

[2] Kennedy TG, Gillio-Meina C, Phang SH (2007). Prostaglandins and the initiation of blastocyst implantation and decidualization.. Reproduction.

[3] Williams CS, Mann M, DuBois RN (1999). The role of cyclooxygenases in inflammation, cancer, and development.. Oncogene.

[4] Garavito RM, DeWitt DL (1999). The cyclooxygenase isoforms: structural insights into the conversion of arachidonic acid to prostaglandins.. Biochim Biophys Acta.

[5] Song H, Lim H, Das SK, Paria BC, Dey SK (2000). Dysregulation of EGF family of growth factors and COX-2 in the uterus during the preattachment and attachment reactions of the blastocyst with the luminal epithelium correlates with implantation failure in LIF-deficient mice.. Mol Endocrinology.

[6] Lim H, Paria BC, Das SK, Dinchuk JE, Langenbach R (1997). Multiple female reproductive failures in cyclooxygenase 2-deficient mice.. Cell.

[7] Attar E, Bulun SE (2006). Aromatase and other steroidogenic genes in endometriosis: translational aspects.. Hum Reprod Update.

[8] McGiff JC, Ferreri NR, Carroll MA (2002). The eicosanoid factor: a determinant of individuality of nephron segments.. J Physiol Pharmacol.

[9] Shah DM (2006). The role of RAS in the pathogenesis of preeclampsia.. Curr Hypertens Rep.

[10] Herse F, Dechend R, Harsem NK, Wallukat G, Janke J (2007). Dysregulation of the circulating and tissue-based renin-angiotensin system in preeclampsia.. Hypertension.

[11] Hsieh YY, Chang CC, Tsai FJ, Hsu CM, Lin CC (2005). Angiotensin I-converting enzyme ACE 2350*G and ACE-240*T-related genotypes and alleles are associated with higher susceptibility to endometriosis.. Mol Hum Reprod.

[12] Xia Y, Wen HY, Kellems RE (2002). Angiotensin II inhibits human trophoblast invasion through AT1 receptor activation.. J Biol Chem.

[13] Braileanu GT, Simasko SM, Speth RC, Daubert DC, Hu J (2002). Angiotensin II increases intracellular calcium concentration in pig endometrial stromal cells through type 1 angiotensin receptors, but does not stimulate phospholipase C activity or prostaglandin F2alpha secretion.. Reprod Fertil Dev.

[14] Liu Z, Armant DR (2004). Lysophosphatidic acid regulates murine blastocyst development by transactivation of receptors for heparin-binding EGF-like growth factor.. Exp Cell Res.

[15] Wang J, Mayernik L, Armant DR (2007). Trophoblast adhesion of the peri-implantation mouse blastocyst is regulated by integrin signaling that targets phospholipase C. Dev Biol.

[16] Klee CB, Crouch TH, Krinks MH (1979). Calcineurin: a calcium- and calmodulin-binding protein of the nervous system.. Proc Natl Acad Sci USA.

[17] Mansuy IM (2003). Calcineurin in memory and bidirectional plasticity.. Biochem Biophys Res Commun.

[18] Martínez-Martínez S, Redondo JM (2004). Inhibitors of the calcineurin/NFAT pathway.. Curr Med Chem.

[19] Crabtree GR, Olson EN (2002). NFAT signaling: choreographing the social lives of cells..

[20] Hogan PG, Chen L, Nardone J, Rao A (2003). Transcriptional regulation by calcium, calcineurin, and NFAT.. Genes Dev.

[21] Liu J, Farmer JD, Lane WS, Friedman J, Weissman I (1991). Calcineurin is a common target of cyclophilin-cyclosporin A and FKBP-FK506 complexes.. Cell.

[22] Iñiguez MA, Martinez-Martinez S, Punzón C, Redondo JM, Fresno M (2000). An essential role of the nuclear factor of activated T cells in the regulation of the expression of the cyclooxygenase-2 gene in human T lymphocytes.. J Biol Chem.

[23] Hernández GL, Volpert OV, Iñiguez MA, Lorenzo E, Martínez-Martínez S (2001). Selective inhibition of vascular endothelial growth factor-mediated angiogenesis by cyclosporin A: roles of the nuclear factor of activated T cells and cyclooxygenase 2.. J Exp Med.

[24] Sugimoto T, Haneda M, Sawano H, Isshiki K, Maeda S (2001). Endothelin-1 induces cyclooxygenase-2 expression via nuclear factor of activated T-cell transcription factor in glomerular mesangial cells.. J Am Soc Nephrol.

[25] Duque J, Fresno M, Iñiguez MA (2005). Expression and function of the nuclear factor of activated T cells in colon carcinoma cells: involvement in the regulation of cyclooxygenase-2.. J Biol Chem.

[26] Canellada A, Ramirez BG, Minami T, Redondo JM, Cano E (2008). Calcium/calcineurin signaling in primary cortical astrocyte cultures: Rcan1–4 and cyclooxygenase-2 as NFAT target genes.. Glia.

[27] Harris RC (2003). Interactions between COX-2 and the renin-angiotensin system in the kidney.. Acta Physiol Scand.

[28] Harris RC, Zhang MZ, Cheng HF (2004). Cyclooxygenase-2 and the renal renin-angiotensin system.. Acta Physiol Scand.

[29] Hernández J, Astudillo H, Escalante B (2002). Angiotensin II stimulates cyclooxygenase-2 mRNA expression in renal tissue from rats with kidney failure.. Am J Physiol Renal Physiol.

[30] Jaimes EA, Tian RX, Pearse D, Raij L (2005). Up-regulation of glomerular COX-2 by angiotensin II: role of reactive oxygen species.. Kidney Int.

[31] Aramburu J, Yaffe MB, López-Rodríguez C, Cantley LC, Hogan PG (1999). Affinity-driven peptide selection of an NFAT inhibitor more selective than cyclosporin A. Science.

[32] Kiani A, García-Cózar FJ, Habermann I, Laforsch S, Aebischer T (2001). Regulation of interferon-gamma gene expression by nuclear factor of activated T cells.. Blood.

[33] Cong J, Diao HL, Zhao YC, Ni H, Yan YQ (2006). Differential expression and regulation of cylooxygenases, prostaglandin E synthases and prostacyclin synthase in rat uterus during the peri-implantation period.. Reproduction.

[34] Kennedy TG, Ross HE (1993). Effect of prostaglandin E2 on rate of decidualization in rats.. Prostaglandins.

[35] Ebert AD, Bartley J, David M (2005). Aromatase inhibitors and cyclooxygenase-2 (COX-2) inhibitors in endometriosis: new questions–old answers?. Eur J Obstet Gynecol Reprod Biol.

[36] Goksu Erol AY, Nazli M, Elis Yildiz S (2011). Expression levels of cyclooxygenase-2, tumor necrosis factor-α and inducible NO synthase in placental tissue of normal and preeclamptic pregnancies..

[37] Shah TJ, Walsh SW (2007). Activation of NF-kappaB and expression of COX-2 in association with neutrophil infiltration in systemic vascular tissue of women with preeclampsia.. Am J Obstet Gynecol.

[38] Howe LR, Subbaramaiah K, Brown AM, Dannenberg AJ (2001). Cyclooxygenase-2: a target for the prevention and treatment of breast cancer.. Endocr Relat Cancer.

[39] Sobolewski C, Cerella C, Dicato M, Ghibelli L, Diederich M (2010). The role of cyclooxygenase-2 in cell proliferation and cell death in human malignancies Int J Cell Biol (doi.

[40] Matsumoto Y, Ishiko O, Deguchi M, Nakagawa E, Ogita S (2001). Cyclooxygenase-2 expression in normal ovaries and epithelial ovarian neoplasms.. Int J Mol Med.

[41] Young JL, Jazaeri AA, Darus CJ, Modesitt SC (2008). Cyclooxygenase-2 in cervical neoplasia: a review.. Gynecol Oncol.

[42] St-Louis I, Singh M, Brasseur K, Leblanc V, Parent S (2010). Expression of COX-1 and COX-2 in the endometrium of cyclic, pregnant and in a model of pseudopregnant rats and their regulation by sex steroids.. Reprod Biol Endocrinol.

[43] Flanagan WM, Corthesy B, Bram RJ, Crabtree GR (1991). Nuclear association of a T-cell transcription factor blocked by FK-506 and cyclosporin A. Nature.

[44] Dechend R, Viedt C, Müller DN, Ugele B, Brandes RP (2003). AT1 receptor agonistic antibodies from preeclamptic patients stimulate NADPH oxidase.. Circulation.

[45] Griendling KK, Berk BC, Socorro L, Tsuda T, Delafontaine P (1988). Secondary signalling mechanisms in angiotensin II-stimulated vascular smooth muscle cells.. Clin Exp Pharmacol Physiol.

[46] Squires PM, Kennedy TG (1992). Evidence for a role for a uterine renin-angiotensin system in decidualization in rats.. J Reprod Fertil.

[47] Sirois J, Richards JS (1993). Transcriptional regulation of the rat prostaglandin endoperoxide synthase 2 gene in granulosa cells. Evidence for the role of a cis-acting C/EBP beta promoter element.. J Biol Chem.

[48] Xie W, Fletcher BS, Andersen RD, Herschman HR (1994). v-src induction of the TIS10/PGS2 prostaglandin synthase gene is mediated by an ATF/CRE transcription response element.. Mol Cell Biol.

[49] Inoue H, Tanabe T (1998). Transcriptional role of the nuclear factor kappa B site in the induction by lipopolysaccharide and suppression by dexamethasone of cyclooxygenase-2 in U937 cells.. Biochem Biophys Res Commun.

[50] Yamamoto K, Arakawa T, Ueda N, Yamamoto S (1995). Transcriptional roles of nuclear factor kappa B and nuclear factor-interleukin-6 in the tumor necrosis factor alpha-dependent induction of cyclooxygenase-2 in MC3T3-E1 cells.. J Biol Chem.

[51] Chen CC, Sun YT, Chen JJ, Chiu KT (2000). TNF-alpha-induced cyclooxygenase-2 expression in human lung epithelial cells: involvement of the phospholipase C-gamma 2, protein kinase C-alpha, tyrosine kinase, NF-kappa B-inducing kinase, and I-kappa B kinase ½ pathway.. J Immunol.

[52] Schroer K, Zhu Y, Saunders MA, Deng WG, Xu XM (2002). Obligatory role of cyclic adenosine monophosphate response element in cyclooxygenase-2 promoter induction and feedback regulation by inflammatory mediators.. Circulation.

[53] Pham H, Chong B, Vincenti R, Slice LW (2008). Ang II and EGF synergistically induce COX-2 expression via CREB in intestinal epithelial cells.. J Cell Physiol.

[54] Corral RS, Iñiguez MA, Duque J, López-Pérez R, Fresno M (2007). Bombesin induces cyclooxygenase-2 expression through the activation of the nuclear factor of activated T cells and enhances cell migration in Caco-2 colon carcinoma cells.. Oncogene.

[55] Xia Y, Wen H, Bobst S, Day MC, Kellems RE (2003). Maternal autoantibodies from preeclamptic patients activate angiotensin receptors on human trophoblast cells.. J Soc Gynecol Investig.

[56] Tabata C, Ogita K, Sato K, Nakamura H, Qing Z (2009). Calcineurin/NFAT pathway: a novel regulator of parturition.. Am J Reprod Immunol.

[57] Derecka K, Sheldrick EL, Wathes DC, Abayasekara DR, Flint AP (2008). A PPAR-independent pathway to PUFA-induced COX-2 expression.. Mol Cell Endocrinol.

[58] Crabtree GR, Clipstone NA (1994). Signal transmission between the plasma membrane and nucleus of T lymphocytes.. Annu Rev Biochem.

[59] Sales KJ, Maldonado-Pérez D, Grant V, Catalano RD, Wilson MR (2009). Prostaglandin F(2alpha)-F-prostanoid receptor regulates CXCL8 expression in endometrial adenocarcinoma cells via the calcium-calcineurin-NFAT pathway.. Biochim Biophys Acta.

[60] Sales KJ, Grant V, Cook IH, Maldonado-Pérez D, Anderson RA (2010). Interleukin-11 in endometrial adenocarcinoma is regulated by prostaglandin F2alpha-F-prostanoid receptor interaction via the calcium-calcineurin-nuclear factor of activated T cells pathway and negatively regulated by the regulator of calcineurin-1.. Am J Pathol.

[61] Cook IH, Evans J, Maldonado-Pérez D, Critchley HO, Sales KJ (2010). Prokineticin-1 (PROK1) modulates interleukin (IL)-11 expression via prokineticin receptor 1 (PROKR1) and the calcineurin/NFAT signalling pathway.. Mol Hum Reprod.

[62] Maldonado-Pérez D, Brown P, Morgan K, Millar RP, Thompson EA (2009). Prokineticin 1 modulates IL-8 expression via the calcineurin/NFAT signaling pathway.. Biochim Biophys Acta.

[63] Page M, Tuckerman EM, Li TC, Laird SM (2002). Expression of nuclear factor kappa B components in human endometrium.. J Reprod Immunol.

[64] Nakamura H, Kimura T, Ogita K, Nakamura T, Takemura M (2004). NF-kappaB activation at implantation window of the mouse uterus.. Am J Reprod Immunol.

[65] Kelly RW, King AE, Critchley HO (2001). Cytokine control in human endometrium.. Reproduction.

[66] Grant KS, Wira CR (2003). Effect of mouse uterine stromal cells on epithelial cell transepithelial resistance (TER) and TNFalpha and TGFbeta release in culture.. Biol Reprod.

[67] Cano E, Canellada A, Minami T, Iglesias T, Redondo JM (2005). Depolarization of neural cells induces transcription of the Down syndrome critical region 1 isoform 4 via a calcineurin/nuclear factor of activated T cells-dependent pathway.. J Biol Chem.

[68] Bradford MM (1976). A rapid and sensitive method for the quantitation of microgram quantities of protein utilizing the principle of protein-dye binding.. Analytical Biochemistry.

